# Impact of Rheumatoid Arthritis and Its Management on Falls, Fracture and Bone Mineral Density in UK Biobank

**DOI:** 10.3389/fendo.2019.00817

**Published:** 2019-11-26

**Authors:** Michael A. Clynes, Karen Jameson, Daniel Prieto-Alhambra, Nicholas C. Harvey, Cyrus Cooper, Elaine M. Dennison

**Affiliations:** ^1^MRC Lifecourse Epidemiology Unit, University of Southampton, Southampton, United Kingdom; ^2^Centre for Statistics in Medicine, University of Oxford, Oxford, United Kingdom; ^3^NIHR Musculoskeletal Biomedical Research Unit, Nuffield Department of Orthopaedics, Rheumatology and Musculoskeletal Sciences, University of Oxford, Oxford, United Kingdom

**Keywords:** Biobank, rheumatoid arthritis, osteoporosis, fracture, fall

## Abstract

**Objectives:** Rheumatoid arthritis (RA) is a systemic chronic inflammatory disease which presents with polyarthritis in addition to extra-articular manifestations. Historically, studies have shown a link between RA and adverse musculoskeletal outcomes but these studies were reported before the widespread use of biologic therapies. The aim of this study was therefore to investigate associations between RA, RA medications and bone mineral density, falls and fractures, using UK Biobank data.

**Methods:** Diagnosis of RA was made using Hospital Episode Statistics (HES) ICD-10 coding. We assessed RA relationships with estimated bone mineral density (eBMD) from heel quantitative ultrasound measurements, self-reported falls (in last year) and HES recorded fracture, adjusted for age, ethnicity, BMI, smoking status, and physical activity.

**Results:** Of 502,543 participants, 3849 (1.4%) of women and 1643 (0.7%) of men had a diagnosis of RA. Median age of the participants was 57 years (IQR 50–63) in women and 58 (IQR 50–64) in men. RA was associated with lower eBMD (men: β −0.244, 95% CI −0.378, −0.110 *p* < 0.001; women: β −0.217, 95% CI −0.297, −0.138 *p* < 0.001) a reported fall in the last year (men: OR 1.54, 95% CI 1.26, 1.87 *p* < 0.001; women: OR 1.36, 95% CI 1.19, 1.56 *p* < 0.001) and fracture in women (OR 1.76, 95% CI 1.43, 2.16 *p* < 0.001). Corticosteroid therapy in men (β −0.934, 95% CI −1.565, −0.304 *p* = 0.004) and disease modifying anti-rheumatic drug (DMARD) use in both sexes (men: β −0.437, 95% CI −0.761, −0.112 *p* = 0.008; women: β −0.243, 95% CI −0.421, −0.065 *p* = 0.007), but not biologic therapy, were associated with a lower eBMD with RA.

**Conclusions:** RA was associated with lower eBMD, increased falls and fracture. Corticosteroid and DMARD therapy, but not biologic therapy, were associated with lower eBMD.

## Introduction

Rheumatoid arthritis (RA) is a chronic systemic inflammatory disease typically presenting with polyarthritis and extra-articular manifestations. Historically, an association between RA and low bone mass (osteoporosis) has been demonstrated; results from several population-based studies have suggested that individuals with RA are at an increased risk of hip fracture (1–5). Furthermore, a study by van Staa and colleagues used data from the British General Practice Research Database to demonstrate that patients with RA have an increased risk of fractures at the hip, pelvis, vertebrae, humerus, and tibia/fibula (6). However, these studies were reported before the widespread use of biologic therapies.

In addition to low bone mass, falls are an important risk factor for fracture. Similarly to low bone mass, historical studies have shown an association between falls risk and RA (7, 8). The increased propensity for falling in individuals with RA is likely to occur secondary to impaired limb coordination, muscle strength, balance and polyarticular pain (9–11).

Despite bone loss being one of the most deleterious consequences of the chronic inflammation seen in RA there are relatively few studies exploring if medications used to treat RA, such as corticosteroids, disease modifying anti-rheumatic drugs (DMARDs), and biologic drugs, have a role in bone protection and these studies have yielded conflicting results (12–14). Cortisone, the first corticosteroid, was the first pharmacological agent used in the treatment of RA in 1949 and offered rapid symptomatic and disease-modifying effects. Corticosteriods remain an extremely effective means of dampening the inflammation associated with RA but are associated with serious long-term side-effects (15). DMARDs are a group of medications which alter the course or outcome of inflammatory conditions and are most commonly used in RA. This group of drugs is recommended as the first-line treatment for RA and include methotrexate, leflunomide, sulfasalazine and hydroxychloroquine, gold (sodium aurothiomalate), azathioprine, ciclosporin and penicillamine (16).Over the past 20 years an increased understanding underlying mechanisms in systemic inflammatory diseases such as RA has permitted the development of potent inhibitors of the inflammatory process termed biologic agents. Biologic agents are drugs that are made from living organisms or their products used in the prevention, diagnosis, or treatment of diseases including RA and cancer. Biologic agents include antibodies, interleukins, and vaccines. Since the introduction of the first biologic agent (Etanercept) in 1998, the treatment of RA has been revolutionized as these therapies can very effectively inhibit inflammation. Biologic agents used in the treatment of RA may have a beneficial effect on bone as we know that pro-inflammatory cytokines such as TNF-α, IL-1, and IL-6 play an important role in bone resorption (17, 18). Common biologic agents used in the treatment of RA include: anti-TNF agents such as Adalimumab, Ertanacept, and Infliximab; Rituximab which targets CD20 on B-cells; Tocilizumab which targets the Anti-IL-6 receptor and more recently janus kinase (JAK) inhibitors such as Baricitinib and Tofacitinib (19).

The UK Biobank is a large prospective cohort established by the UK Medical Research Council and Wellcome Trust as an international resource for the investigation of risk factors for major diseases of middle and older life. The cohort comprises over 500,000 men and women recruited from across the United Kingdom between 2006 and 2010 (20). Using UK National Health Service (NHS) registers, approximately 9.2 million primary invitations were sent to individuals aged 40–69 years living within traveling distance of 22 centers in Great Britain (20, 21). The age range was selected to permit the investigation of the determinants of non-communicable chronic diseases of middle and later life and each individual underwent extensive baseline assessments. The relationship between RA and musculoskeletal health has never previously been explored in such a large cohort of individuals and the UK Biobank affords us the opportunity to explore if the modern management of RA has removed the previously reported association of RA with poor musculoskeletal health. The aims of the current study, therefore, were to use this dataset: (1) to explore if RA is associated with reduced BMD, falls and fracture; and (2) to study how common medications used to treat RA might relate to BMD, falls and fracture risk in individuals with RA, with a particular focus on biologic therapies. The null hypothesis is therefore that RA is not associated with reduced BMD, falls or fracture and that in individuals with RA treatment with corticosteroids, DMARDs and biologic agents is not associated with an improvement in BMD and reduction in falls and fracture risk.

## Methods

### Study Subjects

The study was a cross-sectional analysis using baseline data from the UK Biobank.

### Data Collection

Participants were invited to their regional test center where they completed a series of touchscreen computer-based questionnaires followed by a face-to-face interview with trained research personnel. A transcript of the touchscreen questionnaire can be downloaded (http://www.ukbiobank.ac.uk/wp-content/uploads/2011/06/Touch_screen_questionnaire.pdf?phpMyAdmin=trmKQlYdjjnQIgJ%2CfAzikMhEnx6. Information was collected detailing socio-demographics (age, gender, ethnicity, educational attainment, employment status, household income, and postcode of residence with corresponding deprivation index score), lifestyle factors (including cigarette smoking, diet, physical activity, and alcohol use), current medications and self-reported history of falls in the year prior to the baseline interview.

Estimated bone mineral density (eBMD) was calculated using quantitative heel ultrasound. This was a pragmatic decision, as there was no direct access to dual-energy X-ray absorptiometry (DXA) scanning at all regional test centers. However, heel ultrasound assessed eBMD has been shown to be highly correlated with DXA in previous studies (22, 23). Following calibration and adequate positioning, quantitative heel ultrasound (QUS) was performed on both heels using a Sahara bone sonometer. The recorded measurements included broadband ultrasound attenuation (BUA), speed of sound (SOS), stiffness index, T score (the number of standard deviations from the mean for young individuals of the same sex) and Z score (the number of standard deviations from the mean for individuals of the same sex and age).

A diagnosis of RA was identified in participants from the UK Biobank using HES using ICD-10 codes M05 and M06. A previous history of fracture was identified from HES using ICD-10 codes S02, S12, S22, S32, S42, S52, S62, S72, S82, S92, T02, T08, T10, and T12. HES incorporates data sent by the NHS trusts, clinical commissioning groups and local area teams in England. HES data includes admitted In-patient data from 1997 onwards and this was used in the UK Biobank study. This comprises data on: Admissions and Discharge; diagnostic and operation codes; Maternity; Psychiatric; Critical Care data (supplied to UK Biobank as a separate file). HES records include information on both the NHS-funded admitted patient care and private care within the NHS hospitals. UK Biobank incorporates the critical care data which includes critical care records from April 2006 onwards. More information on the HES data can be found on the HSCIC website (http://www.hscic.gov.uk/hes).

This study was conducted under generic approval from the NHS National Research Ethics Service (17th June 2011, Ref11/NW/0382). Participants provided electronic consent for the baseline assessments.

### Statistical Analysis

Baseline characteristics were analyzed in men and women separately. Descriptive statistics were expressed as medians and interquartile ranges (IQR) for continuous variables and as frequencies (N) and percentages for categorical variables. Multivariate logistic and linear regression models were used to examine the associations between RA and eBMD, falls in the previous year and fracture. The association between RA medications (corticosteroids, DMARDs, and biologics) with eBMD, reported falls and fracture in participants with RA was also explored. Combinations of the aforementioned therapies were also analyzed as combination therapy with DMARDs is often utilized in clinical practice to improve disease control and therefore could potentially influence musculoskeletal health. The regression analyses were undertaken with and without adjustment for the following demographic and lifestyle confounders: age, ethnicity, body mass index (BMI), smoker status, and physical activity. These confounders were selected as they are well-established risk factors for the development of osteoporosis and fracture risk. All analyses were conducted separately for men and women and were performed using Stata v12.1 (Statacorp, College Station, TX, USA).

## Results

### Participant Characteristics

Table 1 shows the summary characteristics. Of 502,543 participants, 3849 (1.4%) of women and 1643 (0.7%) of men were found to have a diagnosis of RA. The median age of the participants was 57 years (IQR 50–63) for women and 58 (IQR 50–64) for men. Most (94.6% of both men and women) of the population were Caucasian. The average Body Mass Index (BMI) of participants was 26.1 kg/m^2^ (IQR 23.5–29.7) for women and 27.3 kg/m^2^ (IQR 25.0–30.1) for men. A higher proportion of men were current smokers [12.6% (*n* = 28612) vs. 9.0% (*n* = 24367)]. 175394 women and 146447 men had heel ultrasound measurements performed generating a mean eBMD T-score of −0.539 and −0.066, respectively. Data were available for self-reported falls in 272089 women and 227785 men, of whom a total of 62818 (23.1%) women and 36284 (15.9%) men had fallen in last year. A total of 14,939 (5.5%) of women and 11,741 (5.1%) men had a history of at least one fracture according to HES between 1997 and their baseline interview.

**Table 1:** Participant demographics.

	**Women**		**Men**		
	**Median**	**IQR**	**Median**	**IQR**	***p*-value**
Age (yrs)	57	50–63	58	50–64	<0.001
Height (cm)	162	158.0–167.0	176	171.0–180.0	<0.001
Weight (kg)	69.1	61.7–78.7	84.3	76.2–93.8	<0.001
BMI (kg/m^2^)	26.1	23.5–29.7	27.3	25.0–30.1	<0.001
Activity (mins/week)^a^	290	150–555	300	150–630	<0.001
Calcium intake (mg/day)^b^	885	668–1146	952	721–1240	<0.001
Alcohol consumption (g/day)^b^	0.0	0.0–16.8	9.8	0.0–36.1	<0.001
	***N***	**%**	***N***	**%**	***p*****-value**
**Smoking status**					<0.001
Never	1,62,067	59.6	1,11,475	49.0	
Previous	85,458	31.4	87,614	38.5	
Current	24,367	9.0	28,612	12.6	
**Ethnicity**					<0.001
White	2,57,456	94.6	2,15,275	94.6	
Fracture^c^	14,939	5.5	11,741	5.1	<0.001
Fallen in last year	62,818	23.1	36,284	15.9	<0.001
RA diagnosis^d^	3,849	1.4	1,643	0.7	<0.001
Taking corticosteroids (RA only)	449	11.7	186	11.3	<0.001
Taking DMARDs (RA only)	1,492	38.8	557	33.9	<0.001
Taking biologics (RA only)	91	2.4	33	2.0	0.011
	**Mean**	**SD**	**Mean**	**SD**	***p*****-value**
Heel ultrasound BMD T-score	−0.539	1.097	−0.066	1.341	<0.001

a Minutes of moderate or vigorous activity in typical week.

b Estimated intake, based on food and beverage consumption the previous day, excluding any supplements.

c From Hospital Episode Statistics using ICD-10 codes S02, S12, S22, S32, S42, S52, S62, S72, S82, S92,T02, T08, T10, T12.

d *From Hospital Episode Statistics using ICD-10 codes M05 and M06*.

Table 2 shows the relationship between falls, eBMD and fracture with RA. A diagnosis of RA was associated with having a lower eBMD in both men (β −0.357, 95% CI −0.439, −0.275 *p* < 0.001) and women (β −0.341, 95% CI −0.385, −0.298 *p* < 0.001). These relationships remained robust following adjustment for age, ethnicity, BMI, smoking status, and physical activity. These confounders were selected as they have been shown to be associated with eBMD and RA in previous studies. Indeed, smoking status constitutes part of the FRAX algorithm for assessing osteoporotic fracture risk (24) and this is consistent with our cohort where the number of cigarettes currently smoked daily was associated with eBMD in both sexes (men: β −0.017, 95% CI −0.018, −0.016 *p* < 0.001; women: OR −0.008, 95% CI −0.009, −0.007 *p* < 0.001). Several studies have shown that exercise is associated with improved BMD (25–27) but in our population the association between hours of moderate or vigorous activity in a typical week and eBMD was small (men: β −0.001, 95% CI −0.001, 0.000 *p* = 0.103; women: OR −0.002, 95% CI −0.003, −0.002 *p* < 0.001). Nevertheless, adjustment for these confounders did not attenuate the association we observed between RA and lower eBMD in both men and women. Individuals with a diagnosis of RA were also significantly more likely to report a fall in the last year than those without (men: OR 2.34, 95% CI 2.11, 2.60 *p* < 0.001; women: OR 1.89, 95% CI 1.77, 2.02 *p* < 0.001). These observations again remained significant after full adjustment for potential confounders. There was also a positive association between a diagnosis of RA and a history of previous fracture (men: OR 1.75, 95% CI 1.47, 2.08 *p* < 0.001; women: OR 2.20, 95% CI 1.99, 2.44 *p* < 0.001) but this association did not remain significant in men following adjustment for confounders (OR 1.14, 95% CI 0.80, 1.64 *p* = 0.463). Table 3 shows that the association between RA and fracture in both sexes remained statistically significant if adjusted for either falls or eBMD and for both falls and eBMD in combination (Table 3).

**Table 2:** Relationship between RA and falls risk, eBMD and fracture risk.

**Men**	**Unadjusted**			**Adjusted**^**a**^		
	**Regression coefficient**	**95% CI**	***p*-value**	**Regression coefficient**	**95% CI**	***p*-value**
Heel ultrasound BMD T-score	−0.357	(−0.439, −0.275)	<0.001	−0.244	(−0.378, −0.110)	<0.001
	**Odds ratio**	**95% CI**	***p*****-value**	**Odds ratio**	**95% CI**	***p*****-value**
Fallen in last year	2.34	(2.11, 2.60)	<0.001	1.54	(1.26, 1.87)	<0.001
Fracture	1.75	(1.47, 2.08)	<0.001	1.14	(0.80, 1.64)	0.463
**Women**	**Unadjusted**			**Adjusted**^**a**^		
	**Regression coefficient**	**95% CI**	***p*****-value**	**Regression coefficient**	**95% CI**	***p*****-value**
Heel ultrasound BMD T-score	−0.341	(−0.385, −0.298)	<0.001	−0.217	(−0.297, −0.138)	<0.001
	**Odds ratio**	**95% CI**	***p*****-value**	**Odds ratio**	**95% CI**	***p*****-value**
Fallen in last year	1.89	(1.77, 2.02)	<0.001	1.36	(1.19, 1.56)	<0.001
Fracture	2.20	(1.99, 2.44)	<0.001	1.76	(1.43, 2.16)	<0.001

a *Adjusted for age, ethnicity, BMI, smoker status and activity*.

**Table 3:** Rheumatoid arthritis as an explanatory variable for fracture, with and without adjustment for number of falls in last year and eBMD.

	***N***	**Odds ratio**	**95% CI**	***p*-value**
**Men**				
Unadjusted	2,29,138	1.75	(1.47, 2.08)	<0.001
Adjusted for falls	2,27,785	1.53	(1.28, 1.82)	<0.001
Adjusted for BMD T-score	1,46,447	1.66	(1.34, 2.05)	<0.001
Adjusted for falls & BMD	1,45,951	1.46	(1.17, 1.81)	0.001
T-score				
**Women**				
Unadjusted	2,73,405	2.20	(1.99, 2.44)	<0.001
Adjusted for falls	2,72,089	2.00	(1.80, 2.21)	<0.001
Adjusted for BMD T-score	1,75,394	1.86	(1.64, 2.12)	<0.001
Adjusted for falls & BMD	1,74,893	1.71	(1.50, 1.94)	<0.001
T-score				

Tables 4, 5 show the association between RA medications (corticosteroids, DMARDs, and biologics) with eBMD, reported falls and fracture in participants with RA. 11.6% (635) of RA participants were taking an oral corticosteroid, 37.3% (2049) were taking a DMARD and 2.3% (124) were taking a biologic. 10.7% (381) of participants with RA were taking a combination of corticosteroids and DMARDs and a small number of participants with RA [21 (0.7%)] were taking corticosteroids, DMARDs and a biologic agent in combination.

**Table 4:** Corticosteroids, DMARDs, and biologics as explanatory variables for T-score, fallen in last year, number of falls in last year and fracture, in female RA participants.

	**Unadjusted**			**Adjusted**^**a**^		
	**Regression coefficient**	**95% CI**	***p*-value**	**Regression coefficient**	**95% CI**	***p*-value**
**CORTICOSTEROIDS**						
Heel ultrasound BMD T-score	−0.371	(−0.527, −0.216)	<0.001	−0.022	(−0.398, 0.353)	0.907
	**Odds ratio**	**95% CI**	***p*****-value**	**Odds ratio**	**95% CI**	***p*****-value**
Fallen in last year	0.91	(0.74, 1.12)	0.370	1.38	(0.84, 2.27)	0.204
Fracture	1.27	(0.95, 1.70)	0.113	1.84	(0.92, 3.67)	0.086
	**Regression coefficient**	**95% CI**	***p*****-value**	**Regression coefficient**	**95% CI**	***p*****-value**
**DMARDs**						
Heel ultrasound BMD T-score	−0.314	(−0.411, −0.217)	<0.001	−0.243	(−0.421, −0.065)	0.007
	**Odds ratio**	**95% CI**	***p*****-value**	**Odds ratio**	**95% CI**	***p*****-value**
Fallen in last year	0.93	(0.81, 1.06)	0.263	0.77	(0.57, 1.03)	0.077
Fracture	0.97	(0.79, 1.20)	0.809	1.02	(0.65, 1.58)	0.945
	**Regression coefficient**	**95% CI**	***p*****-value**	**Regression coefficient**	**95% CI**	***p*****-value**
**BIOLOGICS**						
Heel ultrasound BMD T-score	−0.491	(−0.818, −0.164)	0.003	−0.403	(−1.107, 0.302)	0.262
	**Odds ratio**	**95% CI**	***p*****-value**	**Odds ratio**	**95% CI**	***p*****-value**
Fallen in last year	0.63	(0.39, 1.01)	0.055	0.60	(0.20, 1.82)	0.369
Fracture	1.46	(0.82, 2.61)	0.196	1.19	(0.27, 5.33)	0.817

a *Adjusted for age, ethnicity, BMI, smoker status, and activity*.

**Table 5:** Corticosteroids, DMARDs, and biologics as explanatory variables for T-score, fallen in last year, number of falls in last year and fracture, in male RA participants.

	**Unadjusted**			**Adjusted**^**a**^		
	**Regression coefficient**	**95% CI**	***p*-value**	**Regression coefficient**	**95% CI**	***p*-value**
**CORTICOSTEROIDS**						
Heel ultrasound BMD T-score	−0.285	(−0.567, −0.003)	0.047	−0.934	(−1.565, −0.304)	0.004
	**Odds ratio**	**95% CI**	***p*****-value**	**Odds ratio**	**95% CI**	***p*****-value**
Fallen in last year	1.46	(1.06, 2.01)	0.020	1.64	(0.83, 3.22)	0.155
Fracture	0.85	(0.48, 1.51)	0.586	0.34	(0.05, 2.61)	0.302
	**Regression coefficient**	**95% CI**	***p*****-value**	**Regression coefficient**	**95% CI**	***p*****-value**
**DMARDs**						
Heel ultrasound BMD T-score	−0.431	(−0.612, −0.250)	<0.001	−0.437	(−0.761, −0.112)	0.008
	**Odds ratio**	**95% CI**	***p*****-value**	**Odds ratio**	**95% CI**	***p*****-value**
Fallen in last year	1.19	(0.95, 1.48)	0.123	1.45	(0.95, 2.22)	0.084
Fracture	0.97	(0.67, 1.40)	0.882	1.42	(0.68, 2.98)	0.356
	**Regression coefficient**	**95% CI**	***p*****-value**	**Regression coefficient**	**95% CI**	***p*****-value**
**BIOLOGICS**						
Heel ultrasound BMD T-score	−0.529	(−1.187, 0.128)	0.114	−0.483	(−1.768, 0.803)	0.461
	**Odds ratio**	**95% CI**	***p*****-value**	**Odds ratio**	**95% CI**	***p*****-value**
Fallen in last year	0.85	(0.39, 1.84)	0.678	0.89	(0.18, 4.32)	0.884
Fracture^b^	1.07	(0.32, 3.54)	0.916			

a Adjusted for age, ethnicity, BMI, smoker status and activity.

b *Insufficient number of men with a fracture and on biologics for the adjusted model to run*.

Steroid and DMARD treatment in RA participants was associated with having a lower eBMD in both women (Steroid: β −0.371, 95% CI −0.527, −0.216 *p* < 0.001; DMARD β −0.314, 95% CI −0.411, −0.217 *p* < 0.001) and men (Steroid: β −0.285, 95% CI −0.567, −0.0.003 *p* = 0.047; DMARD β −0.431, 95% CI −0.612, −0.250 *p* < 0.001). These results remained robust following adjustment for confounders with the exception of steroid treatment in women (β −0.022, 95% CI −0.398, −0.353 *p* = 0.907). No significant association was observed between taking biologic medications and eBMD in either men or women following adjustment for confounders although there was a trend toward lower eBMD in both sexes (Tables 4, 5). Following adjustment for confounders there was no significant relationship between any of the RA medications and falls and fracture.

The significant association between DMARDs and eBMD is lost in women post adjustment for confounders when DMARDs are taken in combination with corticosteroids (β −0.067, 95% CI −0.557, −0.424 *p* = 0.789) but remains significant in men (β −0.945, 95% CI −1.799, −0.091 *p* = 0.030). When corticosteroids, DMARDs, and biologic agents are taken by RA participants in combination no significant association is seen with eBMD in either sex.

## Discussion

In this cross-sectional analysis, RA was positively associated with lower eBMD and an increased likelihood of falls and fracture, thus suggesting that despite advances in the management of RA the increased risk of adverse musculoskeletal health persists. RA was associated with fracture even after adjustment for both eBMD and falls, suggesting that some other aspect of bone quality may be impaired in the condition. Furthermore, in this dataset we demonstrated that participants with RA on DMARDs, and male RA participants on corticosteroids, had a significantly lower eBMD; this was not observed with biologics post adjustment for confounders, suggesting that very tight disease control may be beneficial for musculoskeletal health in RA patients. This now needs to be tested in other studies.

In this study we found that 1.4% of women and 0.7% of men in the study population had a diagnosis of RA which is consistent with previous estimations (28) and validates the use of HES data to establish a diagnosis of RA. The association between osteoporosis and RA was previously well-established but the management of RA has enhanced significantly since these studies were performed and much of the data are limited due to either a small number of participants studied or bias in the selection of participants (1, 2, 6, 29, 30). A study by Haugeberg and colleagues sought to address this by establishing a register of patients with RA in Oslo, Norway. Using results from female patients aged between 20 and 70 years and from this register they demonstrated a 2-fold increase in osteoporosis in individuals with RA compared with the general population (31). These findings were, however, contradicted by a previous US population-based study based on the data from the Third National Health and Nutrition Examination Survey (1988–1994) which did not find a difference in femoral neck BMD between RA and non-RA participants (32). The sample size for both these studies was, however, relatively small (394 and 106 RA participants, respectively). The Biobank data afforded us the unique opportunity to estimate eBMD by means of QUS in a much larger cohort (5492) of participants with RA.

The reason for the increased propensity for individuals with RA to develop osteoporosis is likely multifactorial. RA is an inflammatory condition and studies have shown that pro-inflammatory cytokines such as TNF-α, IL-1, and IL-6 play an important role in bone resorption (17, 18). Furthermore, several epidemiological studies have shown positive correlations between osteoporosis and C-reactive protein (CRP) which is a marker of active inflammation (33–38). Additionally, bone loss in RA is further contributed to by the decline in functional capacity and lack of exercise associated with the condition (39).

Our data show that individuals with RA are at a significantly increased risk of falls. This is in concordance with previous studies which have demonstrated a high prevalence of falls (10–54%) in individuals with RA (7, 8, 40–42). The reason for an increased propensity for falling in individuals with RA is again multifactorial. Studies have shown that fall-related risk factors such as impaired heel-toe walking and difficulty standing up without arm use are more common in RA patients, likely secondary to impaired balance and poor lower limb muscle strength (9, 11). In addition, individuals with RA have chronic polyarticular pain which has been shown to be positively related to falls in a cohort of 749 older adults in Boston, USA (10).

Our results are consistent with previous historical studies which have demonstrated that individuals with RA have an increased risk of fracture (1–5) and suggest that despite the modern management of RA an overall increased risk of fracture remains. The positive association between RA and fracture remains robust following adjustment for eBMD and falls. This suggests that neither falls alone or lower eBMD are responsible for the increased fracture risk which is multifactorial in individuals with RA and could be related to aspects of bone quality not measured by QUS which is not addressed in the current study. As the increased risk of fracture in RA participants was not fully attenuated upon adjustment for eBMD this suggests that in clinical practice focusing on improving eBMD alone may not be sufficient to reduce fracture risk and a more holistic approach, including falls prevention, may be of benefit. Indeed, a study by Stanmore and colleagues suggested that in clinical practice RA patients should be asked if they have fallen in the past year to highlight those at high risk of further falls. They suggest that then targeting interventions toward risk factors, such as the use of psychotropic medications and fatigue, could reduce the burden of falls in patients with RA (41).

As bone loss in RA is associated with systemic inflammation, agents which dampen inflammation, and especially newer biologic agents, could theoretically improve bone health and in the current study we also explored whether common medications used to treat RA (corticosteroids, DMARDs, and biologics) modulate the risk of low eBMD, reported falls and fracture we observed in the RA participants. We found that both corticosteroids and DMARDs were associated with a reduced eBMD in RA participants. Steroid use is a well-documented risk factor for the development of osteoporosis in RA patients and our data does support this. Studies have shown that the use of even a small dose of corticosteroids during RA treatment (e.g., prednisone 5 mg/day or equivalent) for more than 3 months, can be associated with fast and persistent bone loss (43). In our study treatment with DMARDs (e.g., methotrexate) was associated with a lower eBMD in participants with RA. To our knowledge the effect of DMARDs on BMD has been little explored but a recent study by Kwon et al. showed no effect of any DMARD on BMD with the exception of leflunomide which was associated with significantly increased vertebral BMD (44). Additionally, a study by Buckley and colleagues showed that at the end of 3 years treatment with low dose methotrexate there was no change in femoral neck or lumbar spine BMD in patients with RA (45).

There was no statistically significant association between biologic therapy and eBMD, falls or fracture for RA participants in our cohort although there was a trend toward lower eBMD. Recent studies have shown that therapies targeting specific cytokines with biologic agents may protect the skeleton though results have been inconsistent (46–50). Few studies have reported a reduction in fractures post initiation with biologic therapies (50). Our findings that steroid treatment in men and DMARD therapy in both sexes is associated with a lower eBMD may reflect a possibility that participants on these medications had more active RA with less tightly controlled inflammation. Corticosteroids in particular are often used in clinical practice to bring an acute flare of synovitis under control. This hypothesis is also supported by our observation that when corticosteroids, DMARDs, and biologics are used in combination, presumably affording a tighter control of inflammation compared to monotherapy, the association with reduced eBMD is lost. This is consistent with previous studies which have shown that in RA patients disease activity and duration are the most important variables regarding the risk of systemic osteoporosis. Some studies have reported such good RA control in all arms that relationships with bone health may be difficult to establish (12). Furthermore, a link has been shown between bone loss in RA and laboratory markers of inflammation such as C-reactive protein and also the development of anti-citrullinated antibodies, a predictor of more severe disease (34, 51). Better RA control may lead to key behaviors associated with better musculoskeletal health. For example, patients may be more physically active, leading to less sarcopenia and falls. The direct effect on bone of control of inflammation may also be relevant (17, 38, 52).

### Limitations

Our study contains strengths and limitations. One of the greatest strengths of this study, and the UK Biobank data in general, is the large sample size which can be included in the analysis. This can, however, lead to small differences between groups reaching the threshold of statistical significance which may not be relevant clinically. We did, however, observe odds ratios of around 2, which suggest biologically relevant relationships. We used QUS for the assessment of eBMD for pragmatic reasons as there was no on site access to DXA (the traditional gold standard) in the UK Biobank research centers but this has been validated in previous studies (23). Indeed, a study by Otter and colleagues demonstrated that heel USS has a 90% sensitivity for a diagnosis of osteoporosis in patients with RA (53). Individuals were self-reporting a history of falls and their medication history and this is therefore subject to recall bias. For medication history in particular, the number of individuals with RA on DMARDs was lower than expected (38.8% of women and 33.9% of men), suggesting that this may have been under-reported or that some of the individuals with a diagnosis of RA but not taking DMARDs may have been misdiagnosed. This may also partially account for the lower eBMD seen individuals with a diagnosis of RA on DMARDs. Confirmation of RA diagnosis via biomarkers may therefore be indicated. It should be noted, however, that although a much higher prevalence of DMARD use (>90%) is reported in cohorts recruited from secondary care settings, this is much lower in data from inception cohorts of patients presenting with inflammatory arthritis in the community (<60%), which is more consistent with our findings (54). Indeed, the response rate for the UK Biobank was only 5.5% and is therefore likely to be subject to a degree of healthy cohort bias, thereby potentially excluding individuals with severe RA disease activity who are more likely to be taking DMARD and biologic medications (55). Only a relatively low number of RA participants [124 (2.3%)] were recorded as being on biologic agents, thus potentially reducing the power to detect associations in this group of participants. Furthermore, the UK Biobank baseline data was accrued between 2006–2010 and in the years subsequent to this it is likely that the proportion of RA patients on biologic agents will have increased. The low prevalence of biologic agent use may therefore reflect a combination of healthy cohort bias and emerging use of these therapies. Interestingly, when we restrict our analysis to those participants who had a recorded diagnosis of RA and also were taking DMARDs, steroids or biologic agents, 3.72% of this sample reported biologics use, more in keeping with modern management. Future studies are therefore indicated to explore these relationships in other cohorts and registries. However, UK Biobank represents an excellent and unique opportunity to explore these issues in a very large data set where eBMD is available. Additionally, a diagnosis of RA and previous fracture was made using HES. If these records were inaccurate or an individual had not attended secondary care in the 9–13 years preceding the study then conceivably the prevalence of RA and fracture may be under-reported. The concordance of prevalence rates with previously reported figures was hence reassuring. The HES data also does not give us any information on disease duration or severity. Finally, the cross-sectional design of the study limits our ability to attribute causation.

In conclusion our study has shown that individuals with RA remain at increased risk of low eBMD, falls and fracture despite advancements in management; that the relationship with fracture was robust to adjustment for eBMD and falls and; that while DMARD and steroid use was associated with poorer bone health, this was not true for biologic therapies.

## Data Availability Statement

The datasets generated for this study will not be made publicly available. Data is available through request via the UK Biobank.

## Ethics Statement

The studies involving human participants were reviewed and approved by NHS National Research Ethics Service (17th June 2011, Ref11/NW/0382). The patients/participants provided their written informed consent to participate in this study.

## Author Contributions

MC wrote and revised the paper and interpreted the data and formulated conclusions. NH and DP-A helped with data interpretation and drafting the paper. KJ analyzed the data. CC and ED provided supervision for the whole project, were involved in data interpretation, and all authors revised the drafted manuscript and approved the final version.

### Conflict of Interest

MC has received support for attending conferences from UCB, Pfizer and Eli Lilly. DP-A's research group has received grants from Amgen, UCB, and Servier, institutional fees for speaker services from Amgen and UCB, and institutional fees for consultancy services from UCB. NH reports personal fees, consultancy, lecture fees and honoraria from Alliance for Better Bone Health, AMGEN, MSD, Eli Lilly, Servier, Shire, UCB, Radius Health, Consilient Healthcare, and Internis Pharma, outside the submitted work. ED has received fees from Pfizer and UCB. CC has received lecture fees and honoraria from Amgen, Danone, Eli Lilly, GSK, Medtronic, Merck, Nestlé, Novartis, Pfizer, Roche, Servier, Shire, Takeda, and UCB outside of the submitted work. The remaining author declares that the research was conducted in the absence of any commercial or financial relationships that could be construed as a potential conflict of interest.

## References

[1] Hooyman JR Melton LJ III Nelson AM O'Fallon WM Riggs BL. Fractures after rheumatoid arthritis. A population-based study. Arthritis Rheum. (1984) 27:1353–61. 10.1002/art.1780271205 6508860

[2] Michel BA Bloch DA Wolfe F Fries JF. Fractures in rheumatoid arthritis: an evaluation of associated risk factors. J Rheumatol. (1993) 20:1666–9. 8295176

[3] Cooper C Coupland C Mitchell M. Rheumatoid arthritis, corticosteroid therapy and hip fracture. Ann Rheum Dis. (1995) 54:49–52. 10.1136/ard.54.1.49 7880122 PMC1005512

[4] Huusko TM Korpela M Karppi P Avikainen V Kautiainen H Sulkava R. Threefold increased risk of hip fractures with rheumatoid arthritis in Central Finland. Ann Rheum Dis. (2001) 60:521–2. 10.1136/ard.60.5.521 11302878 PMC1753630

[5] Orstavik RE Haugeberg G Mowinckel P Hoiseth A Uhlig T Falch JA. Vertebral deformities in rheumatoid arthritis: a comparison with population-based controls. Arch Intern Med. (2004) 164:420–5. 10.1001/archinte.164.4.420 14980993

[6] van Staa TP Geusens P Bijlsma JW Leufkens HG Cooper C. Clinical assessment of the long-term risk of fracture in patients with rheumatoid arthritis. Arthritis Rheum. (2006) 54:3104–12. 10.1002/art.22117 17009229

[7] Armstrong C Swarbrick CM Pye SR O'Neill TW. Occurrence and risk factors for falls in rheumatoid arthritis. Ann Rheum Dis. (2005) 64:1602–4. 10.1136/ard.2004.031195 15817660 PMC1755283

[8] Furuya T Yamagiwa K Ikai T Inoue E Taniguchi A Momohara S. Associated factors for falls and fear of falling in Japanese patients with rheumatoid arthritis. Clin Rheumatol. (2009) 28:1325–30. 10.1007/s10067-009-1229-5 19618097

[9] Durward G Non Pugh C Ogunremi L Wills R Cottee M Patel S. Detection of risk of falling and hip fracture in women referred for bone densitometry. Lancet. (1999) 354:220–1. 10.1016/S0140-6736(99)01871-1 10421309

[10] Leveille SG Jones RN Kiely DK Hausdorff JM Shmerling RH Guralnik JM. Chronic musculoskeletal pain and the occurrence of falls in an older population. JAMA. (2009) 302:2214–21. 10.1001/jama.2009.1738 19934422 PMC2927855

[11] Gaino JZ Bertolo MB Nunes CS Barbosa CM Sachetto Z Davitt M Disease-related outcomes influence prevalence of falls in people with rheumatoid arthritis. Ann Phys Rehabil Med. (2018) 62:84–91. 10.1016/j.rehab.2018.09.003 30278237

[12] Guler-Yuksel M Bijsterbosch J Goekoop-Ruiterman YP de Vries-Bouwstra JK Hulsmans HM de Beus WM. Changes in bone mineral density in patients with recent onset, active rheumatoid arthritis. Ann Rheum Dis. (2008) 67:823–8. 10.1136/ard.2007.073817 17644545

[13] Coulson KA Reed G Gilliam BE Kremer JM Pepmueller PH. Factors influencing fracture risk, T score, and management of osteoporosis in patients with rheumatoid arthritis in the Consortium of Rheumatology Researchers of North America (CORRONA) registry. J Clin Rheumatol. (2009) 15:155–60. 10.1097/RHU.0b013e3181a5679d 19363452

[14] Kim SY Schneeweiss S Liu J Solomon DH. Effects of disease-modifying antirheumatic drugs on nonvertebral fracture risk in rheumatoid arthritis: a population-based cohort study. J Bone Miner Res. (2012) 27:789–96. 10.1002/jbmr.1489 22162140 PMC3725780

[15] Smolen JS Aletaha D McInnes IB Rheumatoid arthritis. Lancet. (2016) 388:2023–38. 10.1016/S0140-6736(16)30173-8 27156434

[16] Allen A Carville S McKenna F. Diagnosis and management of rheumatoid arthritis in adults: summary of updated NICE guidance. BMJ. (2018) 362:k3015. 10.1136/bmj.k3015 30076129

[17] Ding C Parameswaran V Udayan R Burgess J Jones G. Circulating levels of inflammatory markers predict change in bone mineral density and resorption in older adults: a longitudinal study. J Clin Endocrinol Metab. (2008) 93:1952–8. 10.1210/jc.2007-2325 18285417

[18] McLean RR. Proinflammatory cytokines and osteoporosis. Curr Osteoporos Rep. (2009) 7:134–9. 10.1007/s11914-009-0023-2 19968917

[19] Holroyd CR Seth R Bukhari M Malaviya A Holmes C Curtis E The British Society for Rheumatology biologic DMARD safety guidelines in inflammatory arthritis. Rheumatology (Oxford). (2019) 58:220–6. 10.1093/rheumatology/key207 30137623

[20] Harvey NC Matthews P Collins R Cooper C. Osteoporosis epidemiology in UK Biobank: a unique opportunity for international researchers. Osteoporos Int. (2013) 24:2903–5. 10.1007/s00198-013-2508-1 24057481

[21] Ollier W Sprosen T Peakman T. UK Biobank: from concept to reality. Pharmacogenomics. (2005) 6:639–46. 10.2217/14622416.6.6.639 16143003

[22] Taal MW Cassidy MJ Pearson D Green D Masud T. Usefulness of quantitative heel ultrasound compared with dual-energy X-ray absorptiometry in determining bone mineral density in chronic haemodialysis patients. Nephrol Dial Transplant. (1999) 14:1917–21. 10.1093/ndt/14.8.1917 10462271

[23] Hawker GA Jamal SA Ridout R Chase C. A clinical prediction rule to identify premenopausal women with low bone mass. Osteoporos Int. (2002) 13:400–6. 10.1007/s001980200046 12086351

[24] Kanis JA McCloskey EV Johansson H Strom O Borgstrom F Oden A Case finding for the management of osteoporosis with FRAX–assessment and intervention thresholds for the UK. *Osteoporos Int*. (2008) 19:1395–408. 10.1007/s00198-008-0712-1 18751937

[25] Hinton PS Nigh P Thyfault J. Effectiveness of resistance training or jumping-exercise to increase bone mineral density in men with low bone mass: a 12-month randomized, clinical trial. Bone. (2015) 79:203–12. 10.1016/j.bone.2015.06.008 26092649 PMC4503233

[26] Weaver CM Gordon CM Janz KF Kalkwarf HJ Lappe JM Lewis R The National Osteoporosis Foundation's position statement on peak bone mass development and lifestyle factors: a systematic review and implementation recommendations. Osteoporos Int. (2016) 27:1281–386. 10.1007/s00198-015-3440-3 26856587 PMC4791473

[27] Daly RM Dalla Via J Duckham RL Fraser SF Helge EW. Exercise for the prevention of osteoporosis in postmenopausal women: an evidence-based guide to the optimal prescription. Braz J Phys Ther. (2019) 23:170–80. 10.1016/j.bjpt.2018.11.011 30503353 PMC6429007

[28] Symmons D Turner G Webb R Asten P Barrett E Lunt M. The prevalence of rheumatoid arthritis in the United Kingdom: new estimates for a new century. Rheumatology (Oxford). (2002) 41:793–800. 10.1093/rheumatology/41.7.793 12096230

[29] West SG Troutner JL Baker MR Place HM. Sacral insufficiency fractures in rheumatoid arthritis. Spine (Phila Pa 1976). (1994) 19:2117–21. 10.1097/00007632-199409150-00021 7825055

[30] Lane NE Pressman AR Star VL Cummings SR Nevitt MC. Rheumatoid arthritis and bone mineral density in elderly women. The Study of Osteoporotic Fractures Research Group. J Bone Miner Res. (1995) 10:257–63. 10.1002/jbmr.5650100212 7754805

[31] Haugeberg G Uhlig T Falch JA Halse JI Kvien TK. Bone mineral density and frequency of osteoporosis in female patients with rheumatoid arthritis: results from 394 patients in the Oslo County Rheumatoid Arthritis register. Arthritis Rheum. (2000) 43:522–30. 10.1002/1529-0131(200003)43:3<522::AID-ANR7>3.0.CO;2-Y 10728744

[32] Kinjo M Setoguchi S Solomon DH Bone mineral density in older adult patients with rheumatoid arthritis: an analysis of NHANES III. *J Rheumatol*. (2007) 34:1971–5. 17896806

[33] Pasco JA Kotowicz MA Henry MJ Nicholson GC Spilsbury HJ Box JD. High-sensitivity C-reactive protein and fracture risk in elderly women. JAMA. (2006) 296:1353–5. 10.1001/jama.296.11.1353 16985226

[34] Schett G Kiechl S Weger S Pederiva A Mayr A Petrangeli M. High-sensitivity C-reactive protein and risk of nontraumatic fractures in the Bruneck study. Arch Intern Med. (2006) 166:2495–501. 10.1001/archinte.166.22.2495 17159016

[35] Nakamura K Saito T Kobayashi R Oshiki R Oyama M Nishiwaki T. C-reactive protein predicts incident fracture in community-dwelling elderly Japanese women: the Muramatsu study. Osteoporos Int. (2011) 22:2145–50. 10.1007/s00198-010-1425-9 20936400

[36] Ishii S Cauley JA Greendale GA Crandall CJ Danielson ME Ouchi Y. C-reactive protein, bone strength, and nine-year fracture risk: data from the Study of Women's Health Across the Nation (SWAN). J Bone Miner Res. (2013) 28:1688–98. 10.1002/jbmr.1915 23456822 PMC3880424

[37] Dahl K Ahmed LA Joakimsen RM Jorgensen L Eggen AE Eriksen EF. High-sensitivity C-reactive protein is an independent risk factor for non-vertebral fractures in women and men: The Tromso Study. Bone. (2015) 72:65–70. 10.1016/j.bone.2014.11.012 25460573

[38] Cauley JA Barbour KE Harrison SL Cloonan YK Danielson ME Ensrud KE. Inflammatory markers and the risk of hip and vertebral fractures in men: the osteoporotic fractures in men (MrOS). J Bone Miner Res. (2016) 31:2129–38. 10.1002/jbmr.2905 27371811 PMC5240475

[39] Moayyeri A. The association between physical activity and osteoporotic fractures: a review of the evidence and implications for future research. Ann Epidemiol. (2008) 18:827–35. 10.1016/j.annepidem.2008.08.007 18809340

[40] Bohler C Radner H Ernst M Binder A Stamm T Aletaha D. Rheumatoid arthritis and falls: the influence of disease activity. Rheumatology (Oxford). (2012) 51:2051–7. 10.1093/rheumatology/kes198 22879462

[41] Stanmore EK Oldham J Skelton DA O'Neill T Pilling M Campbell AJ. Risk factors for falls in adults with rheumatoid arthritis: a prospective study. Arthritis Care Res (Hoboken). (2013) 65:1251–8. 10.1002/acr.21987 23436687 PMC3881513

[42] Brenton-Rule A Dalbeth N Bassett S Menz HB Rome K. The incidence and risk factors for falls in adults with rheumatoid arthritis: a systematic review. Semin Arthritis Rheum. (2015) 44:389–98. 10.1016/j.semarthrit.2014.08.001 25216947

[43] Grossman JM Gordon R Ranganath VK Deal C Caplan L Chen W. American College of Rheumatology 2010 recommendations for the prevention and treatment of glucocorticoid-induced osteoporosis. Arthritis Care Res (Hoboken). (2010) 62:1515–26. 10.1002/acr.20295 20662044

[44] Kwon OC Oh JS Hong S Lee CK Yoo B Kim YG. Conventional synthetic disease-modifying antirheumatic drugs and bone mineral density in rheumatoid arthritis patients with osteoporosis: possible beneficial effect of leflunomide. Clin Exp Rheumatol. (2019) 37:813–9. 30767868

[45] Buckley LM Leib ES Cartularo KS Vacek PM Cooper SM. Effects of low dose methotrexate on the bone mineral density of patients with rheumatoid arthritis. J Rheumatol. (1997) 24:1489–94. 9263140

[46] Haugeberg G Conaghan PG Quinn M Emery P. Bone loss in patients with active early rheumatoid arthritis: infliximab and methotrexate compared with methotrexate treatment alone. Explorative analysis from a 12-month randomised, double-blind, placebo-controlled study. Ann Rheum Dis. (2009) 68:1898–901. 10.1136/ard.2008.106484 19386610

[47] Hoff M Kvien TK Kalvesten J Elden A Haugeberg G. Adalimumab therapy reduces hand bone loss in early rheumatoid arthritis: explorative analyses from the PREMIER study. Ann Rheum Dis. (2009) 68:1171–6. 10.1136/ard.2008.091264 18801760 PMC2689520

[48] Wijbrandts CA Klaasen R Dijkgraaf MG Gerlag DM van Eck-Smit BL Tak PP. Bone mineral density in rheumatoid arthritis patients 1 year after adalimumab therapy: arrest of bone loss. Ann Rheum Dis. (2009) 68:373–6. 10.1136/ard.2008.091611 18408246 PMC2945478

[49] Eekman DA Vis M Bultink IE Kuik DJ Voskuyl AE Dijkmans BA. Stable bone mineral density in lumbar spine and hip in contrast to bone loss in the hands during long-term treatment with infliximab in patients with rheumatoid arthritis. Ann Rheum Dis. (2011) 70:389–90. 10.1136/ard.2009.127787 20447956

[50] Zerbini CAF Clark P Mendez-Sanchez L Pereira RMR Messina OD Uña CR. Biologic therapies and bone loss in rheumatoid arthritis. Osteoporosis Int. (2017) 28:429–46. 10.1007/s00198-016-3769-2 27796445

[51] de Vries-Bouwstra JK Goekoop-Ruiterman YP Verpoort KN Schreuder GM Ewals JA Terwiel JP Progression of joint damage in early rheumatoid arthritis: association with HLA-DRB1, rheumatoid factor, and anti-citrullinated protein antibodies in relation to different treatment strategies. Arthritis Rheum. (2008) 58:1293–8. 10.1002/art.23439 18438829

[52] Boyle WJ Simonet WS Lacey DL. Osteoclast differentiation and activation. Nature. (2003) 423:337–42. 10.1038/nature01658 12748652

[53] Cryer JR Otter SJ Bowen CJ. Use of quantitative ultrasound scans of the calcaneus to diagnose osteoporosis in patients with rheumatoid arthritis. J Am Podiatr Med Assoc. (2007) 97:108–14. 10.7547/0970108 17369316

[54] Siebert S Lyall DM Mackay DF Porter D McInnes IB Sattar N. Characteristics of rheumatoid arthritis and its association with major comorbid conditions: cross-sectional study of 502 649 UK Biobank participants. RMD Open. (2016) 2:e000267. 10.1136/rmdopen-2016-000267 27403335 PMC4932291

[55] Cook MJ Bellou E Bowes J Sergeant JC O'Neill TW Barton A. The prevalence of co-morbidities and their impact on physical activity in people with inflammatory rheumatic diseases compared with the general population: results from the UK Biobank. Rheumatology (Oxford). (2018) 57:2172–82. 10.1093/rheumatology/key224 30107595 PMC6256331

